# Serum Levels of Interleukin-13 Increase in Subjects with Insulin Resistance but Do Not Correlate with Markers of Low-Grade Systemic Inflammation

**DOI:** 10.1155/2018/7209872

**Published:** 2018-02-21

**Authors:** Camilo P. Martínez-Reyes, Angélica Y. Gómez-Arauz, Israel Torres-Castro, Aarón N. Manjarrez-Reyna, León F. Palomera, Alfonso Olivos-García, Edith Mendoza-Tenorio, Gabriela A. Sánchez-Medina, Sergio Islas-Andrade, Guillermo Melendez-Mier, Galileo Escobedo

**Affiliations:** ^1^Unit of Experimental Medicine, School of Medicine, National University of Mexico, General Hospital of Mexico “Dr. Eduardo Liceaga”, 06726 Mexico City, Mexico; ^2^Departamento de Medicina Experimental, Facultad de Medicina, Universidad Nacional Autónoma de México, 04510 México, DF, Mexico; ^3^Research Division, General Hospital of Mexico “Dr. Eduardo Liceaga”, 06726 Mexico City, Mexico

## Abstract

Experimental evidence in mice suggests a role for interleukin- (IL-) 13 in insulin resistance and low-grade systemic inflammation. However, IL-13 serum levels have not been assessed in subjects with insulin resistance, and associations of IL-13 with parameters of low-grade systemic inflammation are still unknown. Our main goal was to examine the systemic levels of IL-13 in patients with insulin resistance, while also studying the relationship of IL-13 with anthropometric, metabolic, and low-grade systemic inflammatory markers. Ninety-two participants were included in the study and divided into insulin-resistant patients and noninsulin-resistant controls. Blood levels of IL-13, glucose, insulin, triglycerides, cholesterol, tumor necrosis factor-alpha (TNF-*α*), IL-10, proinflammatory (Mon-CD11c^+^CD206^−^), and anti-inflammatory (Mon-CD11c^−^CD206^+^) monocytes, as well as anthropometric parameters, were measured in all volunteers. Insulin-resistant patients showed 2.5-fold higher serum levels of IL-13 than controls (*P* < 0.0001) and significantly increased values of TNF-*α* and Mon-CD11c^+^CD206^−^, with concomitant reductions in IL-10 and Mon-CD11c^−^CD206^+^. Increased IL-13 was extraordinarily well associated with hyperglycemia (*r* = 0.7362) and hypertriglyceridemia (*r* = 0.7632) but unexpectedly exhibited no significant correlations with TNF-*α* (*r* = 0.2907), IL-10 (*r* = −0.3882), Mon-CD11c^+^CD206^−^ (*r* = 0.2745) or Mon-CD11c^−^CD206^+^ (*r* = −0.3237). This study demonstrates that IL-13 serum levels are elevated in patients with insulin resistance without showing correlation with parameters of low-grade systemic inflammation.

## 1. Introduction

Insulin resistance is a key pathophysiological event in the development of type 2 diabetes (T2D), a serious public health problem of global proportions, with alarmingly high morbidity and mortality rates in several countries including USA and Mexico [1, 2]. A growing body of clinical and experimental evidence has consistently shown that insulin resistance is linked to obesity and low-grade systemic inflammation [3, 4]. Specifically, increased body mass index (BMI) and visceral fat accumulation have been shown to augment the risk to develop dyslipidemia, hyperglycemia, and insulin resistance [5]. Low-grade systemic inflammation is characterized by abnormally high serum levels of proinflammatory cytokines (i.e., tumor necrosis factor alpha [TNF-*α*]) and increased percentage of proinflammatory monocytes such as monocytes that exhibit high expression of CD11c and no expression of CD206 (Mon-CD11c^+^CD206^−^) [6, 7]. Low-grade systemic inflammation is also associated with decreased serum concentrations of anti-inflammatory cytokines such as interleukin- (IL-) 10 and low percentages of monocytes exerting anti-inflammatory abilities as is the case of monocytes that show high expression of CD206 and no expression of CD11c (Mon-CD11c^−^CD206^+^) [7, 8]. High amounts of TNF-*α* have been reported to concur with increased adiposity [9], hypertriglyceridemia [8], and impaired insulin sensitivity in adipose and hepatic tissue [10]. Moreover, Saghizadeh and coworkers have previously demonstrated that TNF-*α* is actively expressed in skeletal muscle tissue of insulin-resistant patients as compared to subjects with normal insulin sensitivity [11]. In the same sense, TNF-*α* infusion in healthy individuals is able to induce muscle insulin resistance by increasing phosphorylation of p70 S6 kinase, extracellular signal-regulated kinase −1/2 (ERK-1/2), and c-Jun NH2 terminal kinase (JNK) [12]. Interestingly, phosphorylation of p70 S6 kinase, ERK-1/2, and JNK is associated with decreased activation of the insulin receptor substrate-1 (IRS-1) and Akt substrate 160 [12], which supports the notion that TNF-*α* is a major contributor of insulin resistance in skeletal muscle. On the other side, decreased IL-10 has been related to elevated serum concentrations of TNF-*α*, increased proportion of Mon-CD11c^+^CD206^−^ over the Mon-CD11c^−^CD206^+^ subpopulation, hyperglycemia, and higher levels of insulin resistance in obese subjects [7, 13]. Therefore, low-grade systemic inflammation has now gained increasing attention since it appears to play a causative role in the development of insulin resistance in liver, adipose tissue, and skeletal muscle of obese patients [14].

IL-13 is a cytokine belonging to the alpha-helix protein family that is mainly produced by activated Th2 cells, mast cells, and basophils and has been widely studied in the scenario of helminth parasite infections and allergic asthma [15, 16]. Nevertheless, recent experimental evidence in mice has now found that IL-13 may also participate in low-grade systemic inflammation and insulin resistance [17, 18]. In this sense, it has been reported that exogenous administration of IL-13 improves insulin sensitivity while also decreasing TNF-*α* expression and macrophage infiltration in epididymal adipose tissue of C57BL/6J mice fed a high-fat diet (HFD) [17]. Likewise, a further study demonstrated that IL-13 gene transfer plays a protective role during experimental obesity by diminishing adipocyte hypertrophy, glucose intolerance, insulin resistance, and macrophage infiltration into adipose tissue of HFD-fed C57BL/6J mice [18]. Interestingly, IL-13 has been also associated with improved insulin secretion. In this sense, Darkhal and colleagues previously showed that IL-13 gene overexpression concurs with increased insulin serum levels in mice [18]. Additionally, a recent study demonstrated that IL-13 *in vitro* increases insulin secretion in beta-cells of humans and rats [19], supporting the fact that IL-13 also has an impact on insulin production and release. In parallel, IL-13 has been also shown to play a role in the development of insulin resistance in human beings. In this regard, a previous study demonstrated that IL-13 serum levels are reduced in T2D patients that exhibit increased insulin resistance [20]. However, the role of IL-13 in the pathogenesis of insulin resistance in humans is still unclear, and potential associations of this cytokine with parameters of low-grade systemic inflammation and metabolic dysfunction remain obscure.

The main goal of this work was to examine the systemic levels of IL-13 in patients with insulin resistance, while also studying the relationship of IL-13 with parameters of low-grade systemic inflammation such as TNF-*α*, IL-10, Mon-CD11c^+^CD206^−^ percentage, and Mon-CD11c^−^CD206^+^ percentage, and other insulin resistance-related metabolic markers including fasting blood glucose and insulin, BMI, central obesity, body fat percentage, waist-to-hip ratio, triglycerides, and cholesterol.

## 2. Materials and Methods

### 2.1. Subjects

Ninety-two Mexican adult women and men from the south-central region of Mexico were included in the study. All of the volunteers provided written informed consent, previously approved by the institutional review board of the General Hospital of Mexico, which guaranteed that the study was conducted in accordance with the principles described at the Helsinki Declaration. Volunteers were excluded from the study if they had previous or recent diagnosis of *Diabetes Mellitus*, cardiovascular diseases, chronic renal disease, chronic or acute hepatic disease, blood pressure higher than 135/85 mm Hg, inflammatory or autoimmune disorders, acute or chronic infectious diseases, cancer, and endocrine disorders including hypothyroidism. We also excluded pregnant or lactating women, patients with cardiovascular drug therapy including anti-inflammatory, antiaggregant, and antihypertensive drugs, and subjects without having an overnight fasting of 8–10 hours. All participants enrolled into the study received full medical evaluation, including achievement of clinical history and physical examination by expert physicians.

### 2.2. Insulin Resistance Assessment

The insulin resistance level was estimated by means of calculating the homeostatic model assessment of insulin resistance (HOMA-IR) in each participant. The HOMA-IR value resulted from multiplying fasting insulin concentration (mU/l) by fasting glucose concentration (mmol/l) and then divided by 22.5. Cut-off points for insulin resistance were given according to previous studies validated in the Mexican population [21], as follows: subjects showing HOMA-IR < 3.8 were considered as the control group by having a normal level of insulin resistance. On the contrary, subjects showing HOMA-IR ≥ 3.8 were considered as the insulin-resistant group by having a significant level of insulin resistance.

### 2.3. Anthropometric Measurements

BMI resulted from dividing corporal weight (kg) by height squared (m^2^) and was recorded in all participants, as follows: BMI 18.5–24.9 kg/m^2^, normal weight subjects; BMI 25–29.9 kg/m^2^, overweight subjects; and BMI ≥ 30 kg/m^2^, obese subjects. Central obesity was estimated in each participant by measuring the midpoint between the lower rib margin and iliac crest with a conventional tape in centimeters (cm). Cut-off point values for central obesity were given as follows: women showing 80 cm waist circumference or higher were considered to have central obesity, while men showing 90 cm waist circumference or higher were considered to have central obesity. Body fat percentage was individually estimated by means of using a body composition analyzer (TANITA®, Body Composition Analyzer, Model TBF-300A, Tokyo, Japan).

### 2.4. Metabolic Measurements

Blood samples were collected after overnight fasting and placed into pyrogen-free tubes (Vacutainer™, BD Diagnostics, NJ, USA) at room temperature. Afterwards, collection tubes were centrifuged at 1000*g*/4°C for 25 min and serum samples obtained and stored at −80°C until use. Serum levels of insulin were individually measured in triplicate by means of the Enzyme-Linked ImmunoSorbent Assay (ELISA), following the manufacturer's instructions (Abnova Corporation, Taiwan). Serum levels of glucose were individually measured in triplicate by the glucose oxidase assay, following the manufacturer's instructions (Megazyme International, Ireland). Total cholesterol and triglyceride levels were individually measured in triplicate by enzymatic assays according to the manufacturer's instructions (Roche Diagnostics, Mannheim, Germany). All biochemical parameters were measured at the same time to avoid procedural variations.

### 2.5. Assessment of the Serum Levels of IL-13, TNF-*α*, and IL-10 by ELISA

Blood samples were collected after overnight fasting and placed into pyrogen-free tubes (Vacutainer, BD Diagnostics, NJ, USA) at room temperature. Afterwards, collection tubes were centrifuged at 1000*g*/4°C for 25 min and serum samples obtained and stored at −80°C until use. Serum levels of IL-13, TNF-*α*, and IL-10 were determined in triplicate by ELISA, following the manufacturer's instructions (Peprotech, Mexico). All cytokines were measured at the same time to avoid procedural variations.

### 2.6. Characterization of Monocyte Surface Markers by Flow Cytometry

Blood samples were collected after overnight fasting and placed into pyrogen-free tubes containing EDTA (Vacutainer, BD Diagnostics, NJ, USA). Afterwards, collection tubes were centrifuged at 1800*g*/8°C for 10 min and white blood cells (WBCs) separated using a micropipette. WBCs were separately placed into 1.6 ml pyrogen-free eppendorf tubes containing 1 ml of ACK Lysing Buffer (Life Technologies, USA) and incubated at 4°C for 5 min. Immediately after, cell suspensions were centrifuged at 1800*g*/8°C for 5 minutes and resulting cell pellets washed twice using PBS 1X (Sigma-Aldrich, Mexico). After an extra centrifugation step, supernatants were discarded and resulting cell pellets resuspended in 50 *μ*l of PBS 1X (Sigma-Aldrich, Mexico) for posterior cell counting using trypan blue staining with Neubauer chamber. In each case, 3 *μ*l of Human TruStrain Reagent (BioLegend Inc., USA) was added to 1 × 10^6^ WBCs and then incubated for 7 minutes on ice. Afterwards, each WBC sample was simultaneously incubated with human anti-CD14 PE/Cy7, anti-CD11c PE/Cy5, and anti-CD206/Cy7 APC at 8°C for 20 min in the absence of light. Analysis of the cell surface markers CD11c and CD206 was exclusively performed on CD14-positive cells that correspond to monocytes, using a FACSCanto II flow cytometer (BD Biosciences, Mexico) by means of BD FACSDiva™ software 6.0, acquiring 50,000 events per test in triplicate. PE/Cy7 mouse IgG2, APC/Cy7 mouse IgG1, and PE/Cy5 mouse IgG1 (BioLegend Inc., USA) were used as isotype control antibodies.

### 2.7. Statistical Analysis

Student's *t*-test was used to compare noninsulin-resistant (NIR) and insulin-resistant (IR) subjects in terms of age, HOMA-IR, BMI, waist circumference, body fat percentage, waist-to-hip ratio, fasting blood glucose, serum insulin, systolic blood pressure, total cholesterol, triglycerides, TNF-*α*, IL-10, Mon-CD11c^+^CD206^−^ percentage, and Mon-CD11c^−^CD206^+^ percentage. Student's *t*-test results are expressed as mean ± standard deviation. Pearson's correlation coefficient was calculated for examining the association of IL-13 with anthropometric, metabolic, and systemic inflammation parameters. Pearson's correlation coefficient results are expressed as coefficients (*r*) and *P* values. Differences were considered significant when *P* < 0.05. All statistical analyses were performed using the GraphPad Prism 6.01 software.

## 3. Results

**Figure 1:**
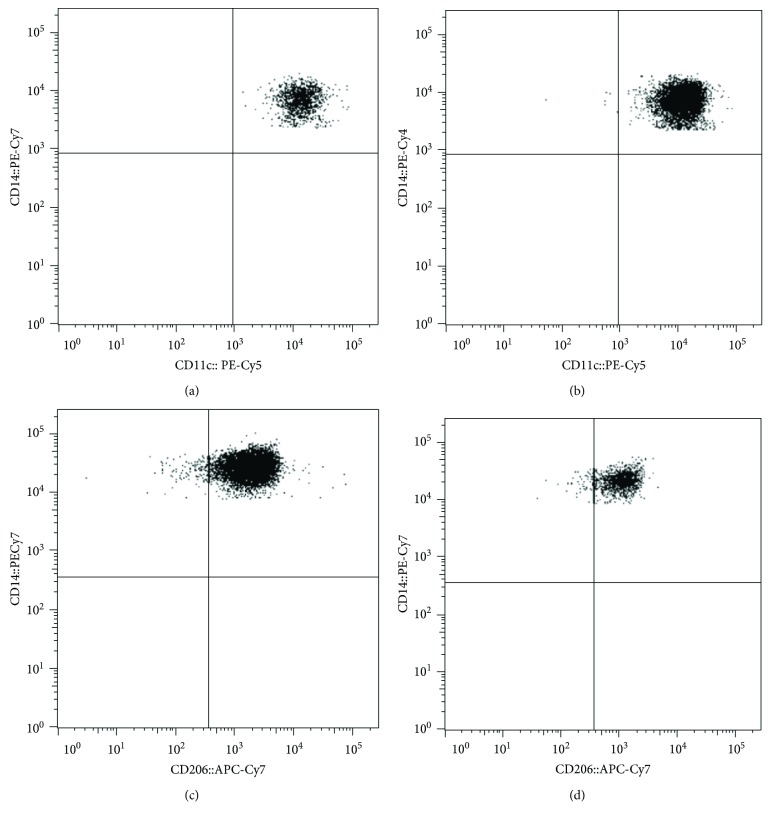
Representative dot plots showing percentages of proinflammatory and anti-inflammatory monocytes in patients with insulin resistance and noninsulin-resistant controls. (a) and (b) illustrate representative flow cytometry dot plots showing percentages of proinflammatory monocytes that express CD11c but do not express CD206 (Mon-CD11c^+^CD206^−^) in controls and insulin-resistant patients, respectively. (c) and (d) illustrate representative dot plots showing percentages of anti-inflammatory monocytes that express CD206 but do not express CD11c (Mon-CD11c^−^CD206^+^) in controls and insulin-resistant patients, respectively. Dot plot quantification can be seen in Table 1.

Ninety-two participants of both sexes were included in the study (47 noninsulin-resistant and 45 insulin-resistant subjects). No significant differences were found in age (for noninsulin-resistant controls mean age 31.02 ± 10.41 years, whereas for insulin-resistant subjects mean age 36.75 ± 11.18 years), woman/man proportion (23 women and 24 men in the noninsulin-resistant group, whereas 22 women and 23 men in the insulin-resistant group), and systolic blood pressure (SBP) (for noninsulin-resistant controls mean systolic pressure 128.40 ± 3.57 mmHg, whereas for insulin-resistant subjects mean systolic pressure 127.9 ± 4.28 mmHg) (Table 1). On the other hand, BMI, waist circumference, and body fat percentage exhibited a significant increase in insulin-resistant subjects as compared to noninsulin-resistant controls (Table 1). Similarly, fasting blood glucose, serum insulin, and HOMA-IR were also higher in the insulin-resistant group than in the noninsulin-resistant group (Table 1). When dyslipidemia was evaluated, triglyceride values showed a clear elevation in insulin-resistant subjects with respect to noninsulin-resistant controls; however, cholesterol levels did not significantly differ between groups (Table 1). In terms of systemic inflammatory parameters, serum levels of TNF-*α* were significantly increased in the insulin-resistant group as compared to noninsulin-resistant individuals, whereas IL-10 was clearly reduced in insulin-resistant subjects with respect to controls (Table 1). At the same time, insulin-resistant subjects showed a significant increase in the proinflammatory monocyte percentage (Mon-CD11c^+^CD206^−^) accompanied by decreasing numbers of monocytes with anti-inflammatory profile (Mon-CD11c^−^CD206^+^) as compared to controls.  Figure 1 shows representative flow cytometry dot plots illustrating the amount of Mon-CD11c^+^CD206^−^ in (A) noninsulin-resistant controls and (B) patients with insulin resistance, as well as the amount of Mon-CD11c^−^CD206^+^ in (C) noninsulin-resistant controls and (D) patients with insulin resistance.

**Figure 2:**
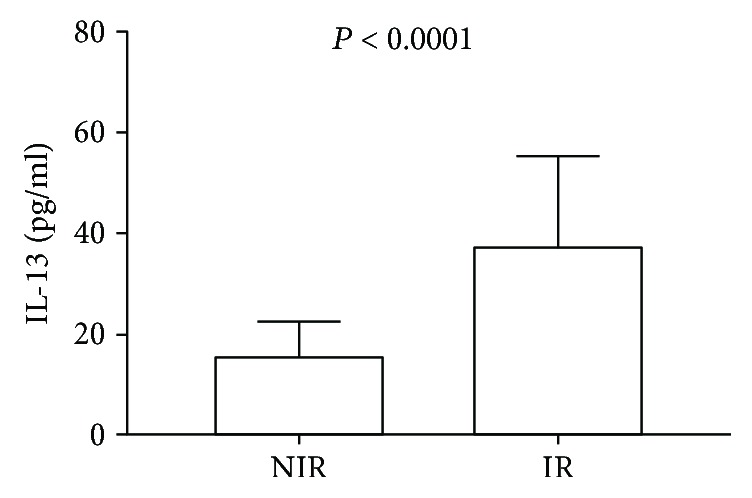
Serum levels of IL-13 in patients with insulin resistance and controls. Systemic levels of IL-13 showed a 2.5-fold significant increase in patients with insulin resistance as compared to noninsulin resistance controls. NIR: noninsulin resistance controls; IR: patients with insulin resistance. A 3.8 cut-off point was used for defining insulin resistance in the study population. Data are expressed as mean ± standard deviation. Differences were considered significant when *P* < 0.05 and calculated using Student's *t*-test.

When IL-13 was examined, we found a significant 2.5-fold increase in the serum levels of IL-13 in insulin-resistant subjects as compared to noninsulin-resistant controls (37.69 ± 17.82 versus 15.88 ± 6.71 pg/ml, resp.) (Figure 2).

**Figure 3:**
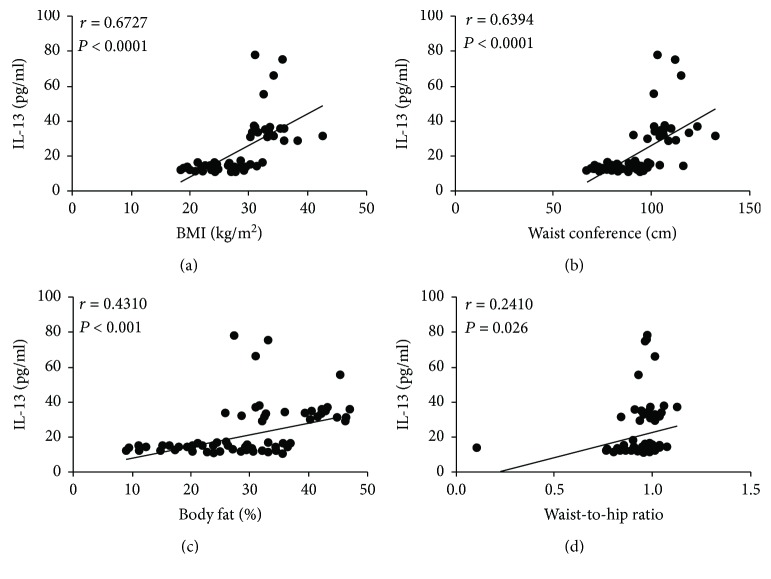
Correlation analysis between IL-13 serum levels and anthropometric parameters in the study population. (a) Correlation analysis between IL-13 serum levels and BMI. (b) Correlation analysis between IL-13 serum levels and waist circumference. (c) Correlation analysis between IL-13 serum levels and body fat percentage. (d) Correlation analysis between IL-13 serum levels and waist-to-hip ratio. Serum levels of IL-13 were moderately associated with BMI and waist circumference and showed to be barely related to body fat percentage and waist-to-hip ratio. BMI: body mass index. Coefficients (*r*) and *P* values were calculated by Pearson's correlation model. The correlation level was considered significant when *P* < 0.05.

IL-13 was significantly elevated in the insulin-resistant group. Therefore, our next step was to identify anthropometric, metabolic, and inflammatory parameters that were clearly related to the elevation in the serum concentrations of this cytokine. IL-13 exhibited a strong positive correlation with BMI (*r* = 0.6727, *P* < 0.0001) and waist circumference (*r* = 0.6394, *P* < 0.0001) (Figures 3(a) and 3(b), resp.). Moreover, serum levels of IL-13 were moderately associated with body fat percentage (*r* = 0.4310, *P* < 0.001) and waist-to-hip ratio (*r* = 0.2410, *P* = 0.026) (Figures 3(c) and 3(d), resp.).

**Figure 4:**
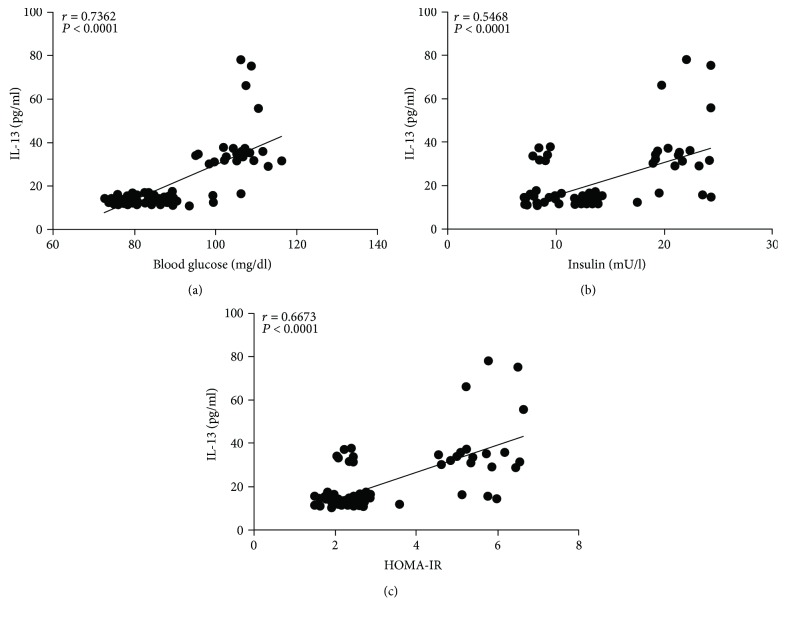
Correlation analysis between IL-13 serum levels and parameters of glucose metabolism in the study population. (a) Correlation analysis between IL-13 serum levels and blood glucose. (b) Correlation analysis between IL-13 serum levels and insulin. (c) Correlation analysis between IL-13 serum levels and HOMA-IR value. Serum levels of IL-13 were strongly associated with blood glucose and showed to be moderately related to insulin and HOMA-IR value. HOMA-IR, homeostatic model assessment of insulin resistance. Coefficients (*r*) and *P* values were calculated by Pearson's correlation model. The correlation level was considered significant when *P* < 0.05.

**Figure 5:**
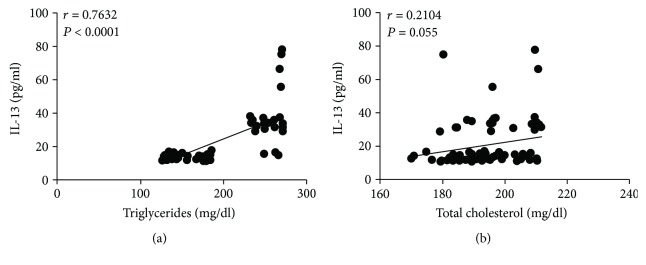
Correlation analysis between IL-13 serum levels and parameters of lipid metabolism in the study population. (a) Correlation analysis between IL-13 serum levels and triglycerides. (b) Correlation analysis between IL-13 serum levels and total cholesterol. Serum levels of IL-13 were strongly associated with blood triglycerides but showed no significant correlation with cholesterol. Coefficients (*r*) and *P* values were calculated by Pearson's correlation model. The correlation level was considered significant when *P* < 0.05.

In the specific case of metabolic parameters, serum IL-13 showed a strong positive relationship with fasting blood glucose (*r* = 0.7362, *P* < 0.0001) and HOMA-IR (*r* = 0.6673, *P* < 0.0001) (Figures 4(a) and 4(c), resp.). Despite being significant, statistical correlation between IL-13 and serum insulin was positive but tended to be moderate (*r* = 0.5468, *P* < 0.0001) (Figure 4(b)). Furthermore, IL-13 was strongly related to the amount of triglycerides (*r* = 0.7632, *P* < 0.0001) but showed no significant correlation with total cholesterol (*r* = 0.2104, *P* = 0.055) (Figures 5(a) and 5(b), resp.).

**Figure 6:**
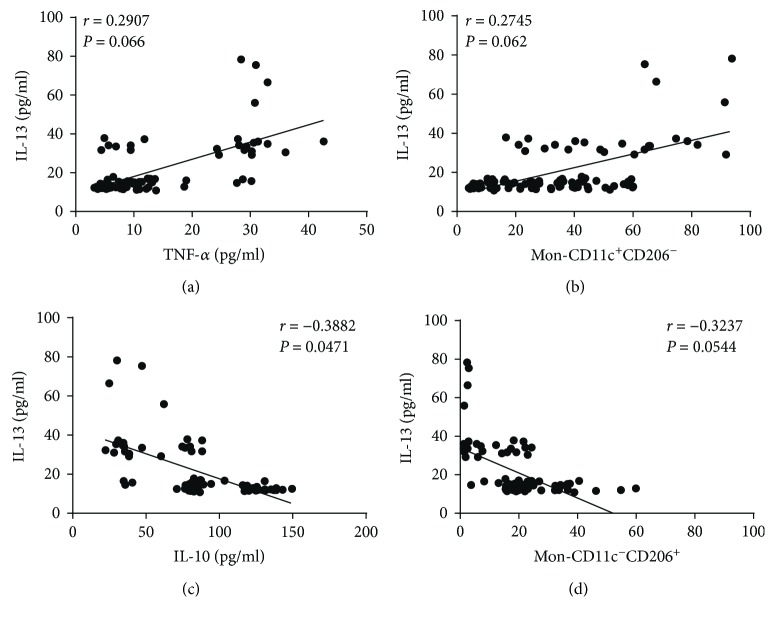
Correlation analysis between IL-13 serum levels and parameters of low-grade systemic inflammation in the study population. (a) Correlation analysis between IL-13 serum levels and circulating concentration of TNF-*α*. (b) Correlation analysis between IL-13 serum levels and the percentage of proinflammatory monocytes Mon-CD11c^+^CD206^−^. (c) Correlation analysis between IL-13 serum levels and circulating concentration of IL-10. (d) Correlation analysis between IL-13 serum levels and the percentage of anti-inflammatory monocytes Mon-CD11c^−^CD206^+^. Serum levels of IL-13 were barely associated with IL-10 but showed no significant correlations with TNF-*α*, Mon-CD11c^+^CD206^−^, and Mon-CD11c^−^CD206^+^. Mon-CD11c^+^CD206^−^, proinflammatory monocytes that express CD11c but do not express CD206; Mon-CD11c^−^CD206^+^, anti-inflammatory monocytes that express CD206 but do not express CD11c. Coefficients (*r*) and *P* values were calculated by Pearson's correlation model. The correlation level was considered significant when *P* < 0.05.

In terms of systemic inflammatory markers, circulating levels of IL-13 exhibited no significant correlations with serum TNF-*α* (*r* = 0.2907, *P* = 0.066) or Mon-CD11c^+^CD206^−^ monocyte percentage (*r* = 0.2745, *P* = 0.062), both of them typical proinflammatory parameters (Figures 6(a) and 6(b), resp.). On the contrary, IL-13 was barely correlated with IL-10 serum levels (*r* = −0.3882, *P* = 0.0471) but showed no significant association with Mon-CD11c^−^CD206^+^ monocyte percentage (*r* = −0.3237, *P* = 0.0544), which have been shown to exert anti-inflammatory actions (Figures 6(c) and 6(d), resp.).

## 4. Discussion

IL-13 is a cytokine that belongs to the alpha-helix protein family and is mainly produced by Th2-activated T lymphocytes, mast cells, and basophils [22]. Besides having been shown to play a critical role in helminth parasite infections and allergic asthma [13, 14], IL-13 has been now suggested to exert additional functions in the development of metabolic alterations such as insulin resistance and hyperglycemia [17, 18]. However, clinical findings in humans regarding the role of IL-13 in metabolic disease are still controversial.

In this sense, it has been previously shown that serum levels of IL-13 are significantly reduced in type 2 diabetic patients with coronary artery disease as compared to healthy controls [23]. Similarly, a recent study demonstrated that type 2 diabetic patients show decreased serum levels of IL-13 with respect to normal-glucose tolerant individuals [20]. On the contrary, it has been also reported that morbidly obese patients with insulin resistance exhibit higher values of serum IL-13 than normal weight controls, and bariatric surgery was able to reduce IL-13 serum concentrations after 1-year of the surgical procedure [24]. Likewise, expression of interleukin-13 receptor subunit alpha-2 (IL-13RA2) has been demonstrated to increase in peripheral blood mononuclear cells (PBMC) of obese children with abnormal insulin sensitivity as compared to normal weight boys [25]. This apparent contradiction could be attributed to the possible role of IL-13 in the insulin resistance pathogenesis that involves the liver, adipose tissue, skeletal muscle, and pancreatic beta-cells. In this regard, a previous study in mice showed that IL-13 gene deficiency concurs with reduced phosphorylation of IRS-1 and AKT in liver, adipose tissue, and skeletal muscle, which was directly related to decreased insulin sensitivity in the aforementioned tissues [26]. Interestingly, IRS-1 and AKT phosphorylation was found to depend on the activation of the signal transducer and activator of transcription (STAT) 3 and STAT6 [26], a well-known family of transcription factors with the ability to elicit anti-inflammatory signaling pathways in response to IL-13. Also, IL-13 has been shown to promote insulin secretion in pancreatic beta-cells [19], which is associated with compensatory hyperinsulinemia aimed to counteract hyperglycemia and insulin resistance. Then, it is reasonable to speculate that IL-13 plays a protective role in insulin resistance by promoting IRS-1 and AKT phosphorylation in insulin-dependent tissues via STAT3 and STAT6 activation as well as improvement of the beta-cell function. However, together with other studies [17, 24, 25], our work shows a significant increase in the serum levels of IL-13 in insulin-resistant patients, which appears to disagree with previous evidence. In this sense, a previous work showed that IL-13 serum levels significantly increase as the severity of T2D-related chronic heart failure also increases [27]. Similarly, a very recent study demonstrated that IL-13 gene expression tended to increase in the left ventricular free wall of T2D patients with heart failure as compared to healthy donors [28]. Interestingly, despite IL-13 gene tended to be upregulated, the IL-13 receptor subunit alpha 1 (IL-13R*α*1) production was significantly decreased in the same cardiac muscle specimens of T2D patients with heart failure, who are by definition insulin-resistant [28]. In some extent, this information is consistent with our findings and suggests a progressive loss of the cellular capacity to respond to IL-13 in the scenario of insulin resistance. Such a state of “IL-13 action resistance” may partially explain the increment of the IL-13 serum levels in several cohorts of patients with insulin resistance, including our own study population. To the best of our knowledge, this is one of the first studies suggesting a state of IL-13 action resistance, characterized by high levels of IL-13, reduced activation of the IL-13-dependent signaling pathway in insulin-dependent tissue, and consequently increased insulin resistance. Nevertheless, we have drawn a speculative hypothesis to explain the apparently contradictory results regarding the role of IL-13 in the development of insulin resistance, and the discussion of this information makes no attempt to conjecture beyond that. For this reason, it is still of enormous importance to study the role of IL-13 in the pathogenesis of insulin resistance, also evaluating the possible existence of a state of IL-13 action resistance in patients with altered insulin sensitivity.

In our study, serum concentrations of IL-13 exhibited a strong correlation with obesity-related anthropometrical parameters including BMI and central obesity. As it has been previously reported, increased BMI and central obesity are key contributing factors to the development of insulin resistance, metabolic syndrome, and type 2 diabetes [29]. Central obesity directly results from the expansion of white adipose cells that accumulate around the viscera of the abdominal cavity [30]. Interestingly, it has been recently shown that increased central obesity and body weight are associated with elevation in the circulating levels of IL-13 [31]. Furthermore, Kwon and coworkers demonstrated that IL-13 is overproduced in the white adipose tissue of HFD-fed mice and obese humans while also reported that adipocytes were the main cellular source of this cytokine [17]. These findings may explain the extremely strong association observed in our study between serum IL-13 levels and obesity-related anthropometrical parameters such as central obesity and, in some extent, BMI. In other words, our data confirm a direct link between fat mass expansion and IL-13 overproduction, which could be supported by the fact that hypertrophic and hyperplasic adipocytes increase their ability to synthesize IL-13. However, we only studied the relationship of obesity-related anthropometrical parameters with IL-13 serum concentrations by means of statistical correlation models and the discussion of these results makes no attempt to conjecture beyond that. Further research is needed to draw conclusions regarding the capacity of white adipose cells to release IL-13 into the bloodstream and the potential role of this cytokine in the development of insulin resistance in obese patients.

Another phenomenon captured in our study is that IL-13 serum levels appear to be extraordinarily correlated with elevated blood values of glucose in the study population, and especially in subjects with insulin resistance. Consistent with our results, Nehete and coworkers previously demonstrated that obese chimpanzees show increased serum levels of IL-13, glucose, and glucagon, a peptide hormone in charge of raising glucose concentration in the bloodstream [32]. This finding suggests a direct link among IL-13, glucagon, and elevated glucose levels. In this sense, glucagon-like peptide-1 (GLP-1) is a peptide hormone able to reduce glucose levels by restricting the secretion of glucagon [33]. Recently, GLP-1 has been also shown to decrease IL-13 production in LPS-treated human eosinophils [34], which supports the idea that increased serum IL-13 could be directly associated with elevated blood glucose levels via glucagon-dependent mechanisms. Nevertheless, it is important to note that IL-13 has been also demonstrated to downregulate the hepatic production of glucose in mice [26] and increase glucose uptake in skeletal muscle cells *in vitro* [20], which seems to disagree with our results. We want to speculate that such a discrepancy could be explained by the fact that protective effects of IL-13 depend on reaching a critical concentration, in which high IL-13 levels are able to counteract the elevation of blood glucose and insulin resistance. In this hypothetical scenario, it is expected to find a positive correlation between IL-13 and insulin, a hormone widely known to counteract the effects of glucagon and decrease glucose levels. Interestingly, our results show a positive correlation between increased IL-13 and elevated values of serum insulin. Consistent with this hypothesis, it has been recently demonstrated that IL-13 promotes beta-cell survival and insulin secretion in human pancreatic beta-cell *in vitro* cultures [19]. However, it is still of great importance to examine the possible effect of IL-13 in regulating glucagon, GLP-1, insulin, and blood glucose levels with the aim of merging apparently controversial data regarding the role of this cytokine in glucose metabolism.

Numerous studies have suggested a direct relationship between IL-13 and lipid metabolism, especially in the scenario of metabolic dysfunction. For instance, a recent work demonstrated that PBMC show increased IL-13 expression in type 2 diabetic nephropathy patients that also exhibit hypertriglyceridemia [35]. Similarly, overweight and obese pregnant women with gestational hypertension show increased IL-13 concentration, which in turn is associated with elevated triglyceride levels [36]. Moreover, metabolic syndrome subjects exhibit increased serum IL-13 that is also correlated with rising concentration of blood sugar and triglycerides [37]. Our data expand on this body of work by revealing that circulating levels of IL-13 are strongly correlated with triglyceride values in insulin-resistant individuals. Interestingly, Tsao and coworkers previously demonstrated that IL-4, another Th2 cytokine with a similar structure and function to IL-13, is able to induce lipolysis in 3T3L1 adipocytes, thus increasing glycerol release and secretion into the culture supernatant [38]. This work concurs with our findings and reveals a novel function of Th2 cytokines in the regulation of triglyceride metabolism; however, there is no evidence yet exploring whether IL-13 may have a similar effect to IL-4. Further research is needed to evaluate the possible role of IL-13 in lipid metabolism and identify novel molecular targets with the aim of reducing triglyceride levels and cardiovascular risk in patients with insulin resistance.

Besides examining its association with insulin resistance-related metabolic markers, IL-13 was also studied in terms of low-grade systemic inflammation. As described above, low-grade systemic inflammation is hallmarked by increased levels of proinflammatory cells and cytokines whereby immune cells and cytokines with anti-inflammatory actions are decreased [39]. Consistent with this notion, we saw a clear elevation in the circulating levels of TNF-*α* and proinflammatory monocytes Mon-CD11c^+^CD206^−^ in insulin-resistant patients with respect to noninsulin-resistant controls. Simultaneously, circulating levels of IL-10 and anti-inflammatory monocytes Mon-CD11c^−^CD206^+^ were also significantly diminished in patients with insulin resistance. However, this state of low-grade systemic inflammation did not relate to serum IL-13, although several reports suggest an association of IL-13 with proinflammatory and anti-inflammatory immune responses [17, 18, 40, 41]. In this sense, it has been previously reported that IL-13 does not always correlate with low-grade systemic inflammation parameters. In fact, a recent study conducted in morbidly obese men with metabolic syndrome showed a significant increase in the serum levels of IL-6 and IL-12, both cytokines with proinflammatory actions, without reporting any difference in IL-13 [42]. Similarly, TNF-*α* soluble receptor levels were shown to raise in plasma of burn-induced systemic inflammatory response syndrome children whereas IL-13 serum levels remained unchanged [43]. Furthermore, Matia-García and coworkers recently reported that young obese subjects with hypertriglyceridemia exhibit low-grade systemic inflammation characterized by increasing levels of C-reactive protein (CRP) and IL-6, accompanied by reduced IL-10 serum concentration [44]. Interestingly, IL-13 showed neither statistical changes between obese and normal weight subjects nor significant correlation coefficients with CRP, IL-6, and IL-10 [44]. Therefore, our results reveal that serum levels of IL-13 elevate in insulin resistance without showing correlation with markers of low-grade systemic inflammation in humans.

Finally, it is important to note that serum IL-13 levels appear to be grouped in two main clusters, characterized by high and low production of this cytokine. Notably, ninety-five percent of low-IL-13 producers showed a HOMA-IR value below 3, whereas an inverse tendency was seen in high-IL-13 producers that exhibited increasing levels of insulin resistance (HOMA-IR > 4.5). Moreover, ninety percent of high-IL-13 producers had central obesity and hyperglycemia, while a similar amount also showed triglyceride levels higher than 200 mg/dl (data not shown), which suggests that increased IL-13 could concur with the development of metabolic syndrome. However, we did not categorize the study subjects according to the number of metabolic syndrome risk factors and the discussion of these results makes no attempt to conjecture beyond that. Further clinical research is needed to understand whether low and high-IL-13 producers have different risks for developing metabolic syndrome and its cardiovascular comorbidities.

## 5. Conclusions

This study demonstrates that serum levels of IL-13 are significantly elevated in insulin-resistant patients without showing correlation with parameters of low-grade systemic inflammation such as TNF-*α*, IL-10, and monocytes that show expression of CD11c and CD206. Hyperglycemia and hypertriglyceridemia appear to be strongly linked to the increase in IL-13, which suggest a novel role of this cytokine in the regulation of glucagon-dependent pathways and lipolysis that should be addressed in patients at higher cardiovascular risk such as the vast majority of individuals living with insulin resistance, metabolic syndrome, and T2D. Current results also allow us to speculate regarding the existence of a state of IL-13 action resistance that could be associated with increased serum IL-13 levels in insulin-resistant patients, a notion that needs to be elucidated in basic and clinical research studies. The study of IL-13 in the development of insulin resistance may provide novel insights regarding the role of cytokines in the pathogenesis of metabolic disease and immune hyperactivation. We also remark the urgency of performing clinical studies evaluating whether IL-13 may represent a novel risk marker of insulin resistance in human beings.

## Figures and Tables

**Table 1:** Demographic, anthropometric, metabolic, and immunological characteristics of the study subjects.

Parameters	NIR	IR	*P*
Gender (W/M)	23/24	22/23	n.s.
Age (years)	31.02 ± 10.41	36.75 ± 11.18	n.s.
BMI (kg/m^2^)	26.82 ± 4.89	32.92 ± 2.34	<0.001
Waist circumference (cm)	86.87 ± 13.00	105.00 ± 6.21	<0.001
Body fat (%)	26.61 ± 8.15	36.99 ± 7.42	<0.001
Waist-to-hip ratio	0.92 ± 0.12	0.97 ± 0.04	n.s.
SBP (mmHg)	128.4 ± 3.57	127.9 ± 4.28	n.s.
Blood glucose (mg/dl)	84.17 ± 9.79	104.9 ± 5.58	<0.0001
Serum insulin (mU/l)	11.16 ± 2.38	21.56 ± 1.89	<0.0001
HOMA-IR	2.28 ± 0.39	5.59 ± 0.64	<0.0001
Total cholesterol (mg/dl)	193.7 ± 10.28	198.0 ± 10.18	n.s.
Triglycerides (mg/dl)	159.40 ± 37.93	256.6 ± 13.19	<0.0001
TNF-*α* (pg/ml)	8.26 ± 3.45	30.29 ± 3.93	<0.0001
IL-10 (pg/ml)	100.8 ± 26.41	37.04 ± 10.36	<0.0001
Mon-CD11c^+^CD206^−^ (%)	28.30 ± 17.18	60.93 ± 20.68	<0.001
Mon-CD11c^−^CD206^+^ (%)	24.23 ± 9.46	6.05 ± 5.71	<0.0001

Data are expressed as mean ± standard deviation. Significant differences were estimated by means of performing two-way Student's *t*-test. Differences were considered significant when *P* < 0.05. W: women; M: men; BMI: body mass index; SBP: systolic blood pressure; HOMA-IR: homeostatic model assessment of insulin resistance; TNF-*α*: tumor necrosis factor alpha; IL: interleukin; Mon CD11c^+^CD206^−^: proinflammatory monocytes; Mon CD11c^−^CD206^+^: anti-inflammatory monocytes; n.s.: nonsignificant differences.
